# Resuscitation in paediatric septic shock using vitamin C and hydrocortisone (RESPOND): The RESPOND randomised controlled trial statistical analysis plan

**DOI:** 10.1016/j.ccrj.2026.100183

**Published:** 2026-06-16

**Authors:** Kristen S. Gibbons, Sainath Raman, Lalitha AV, Marino Festa, Shane George, Renate Le Marsney, Paula Lister, Debbie A. Long, Karthi Nallasamy, Anton Pak, Daniela Carla de Souza, Barbara Zangerl, Luregn J. Schlapbach, the Resuscitation in Paediatric Septic Shock using Vitamin C and Hydrocortisone (RESPOND) Study Group and the Australian and New Zealand Intensive Care Society Paediatric Study Group (ANZICS PSG)

**Affiliations:** a Children's Intensive Care Research Program, Child Health Research Centre, Faculty of Medicine, The University of Queensland, Brisbane, QLD, Australia; b Paediatric Intensive Care Unit, Queensland Children's Hospital, South Brisbane, QLD, Australia; c Paediatric Intensive Care Unit, St. John's Medical College, Bangalore, India; d Kids Critical Care Research, Paediatric Intensive Care Unit, Westmead Children's Hospital, Sydney, NSW, Australia; e Paediatric Critical Care Unit, Gold Coast University Hospital, Gold Coast, Queensland, Australia; f Paediatric Critical Care Unit, Sunshine Coast University Hospital, Sunshine Coast, Queensland, Australia; g School of Medicine and Dentistry, Griffith University, Southport, QLD, Australia; h School of Nursing, Centre for Healthcare Transformation, Queensland University of Technology, Brisbane, Queensland, Australia; i Paediatric Critical Care Unit, Advanced Paediatrics Centre, Post Graduate Institute of Medical Education and Research, Chandigarh, India; j Centre for the Business and Economics of Health, The University of Queensland, Saint Lucias, Queensland, Australia; k Paediatric Intensive Care Unit, Hospital Universitário da Universidade de São Paulo, Sao Paolo, Brazil; l Department of Intensive Care and Neonatology, and Children's Research Center, University Children's Hospital Zurich, University of Zurich, Zurich, Switzerland

**Keywords:** Vitamin C, Critically ill, Child, Septic shock, Clinical trial

## Abstract

**Background:**

The Resuscitation in Paediatric Septic Shock using Vitamin C and Hydrocortisone (RESPOND) trial is a multicentre randomised controlled trial exploring whether the use of hydrocortisone alone, or in combination with vitamin C, increases time alive and free of vasopressors for critically ill children.

**Objective:**

To present the prespecified statistical analysis plan (SAP) for the RESPOND trial prior to finalising recruitment and locking the trial dataset.

**Design, setting, and participants:**

The RESPOND trial is a three-arm, parallel group, open-label, randomised controlled trial, recruiting in paediatric intensive care units in Australia, New Zealand, India, and Brazil. The planned sample size is 384 participants.

**Main outcome measures:**

The primary outcome is time alive and free of inotropes/vasopressors, censored at 7 days post-randomisation. Secondary outcomes include clinical (e.g. alive and free of multi-organ dysfunction, length of stay), safety, health economics (e.g. incremental costs, quality-adjusted life years), and long-term outcomes (measured at 6 months post-randomisation; e.g. health-related quality of life).

**Results and conclusions:**

The SAP was designed by the Chief Investigators and approved by the RESPOND Steering Committee. Statistical analyses are summarised. The primary outcome will be analysed using quantile regression adjusted for stratification variables. Appropriate statistical comparisons between groups were planned and described in a way that is transparent, available to the public, verifiable, and predetermined before completion of data collection. The trial statistician, RESPOND Steering Committee members, and SAP authors remain blind to treatment allocation throughout the study. Data Safety and Monitoring Board members were provided with safety data with masked group identifiers during interim analyses. The RESPOND trial commenced recruitment in December, 2021, and aims to complete recruitment by mid-2026.

**Trial registration:**

ACTRN12621000247875.

## 1 Introduction

Sepsis—defined as organ dysfunction secondary to an infection—affects millions of children worldwide each year and results in significant morbidity and mortality.[1], [2], [3] While improvements in healthcare have resulted in a gradual reduction in mortality^4^ over the last few decades, interventions delivered as part of acute resuscitation have had limited impact on outcomes.^5^

Most recommendations for children in the Surviving Sepsis Campaign guidelines lack high-grade evidence,^6^ and evidence is slow to accrue in paediatric intensive care, with less than a quarter of paediatric intensive care trials recruiting >100 patients.^6^ To date, evidence on adjunctive therapies for children with septic shock remains scarce. For example, while the adult literature report equivocal benefit from intravenous vitamin C^7^, paediatric evidence is lacking apart from pilot studies.^8^^,^^9^ It is hypothesised that, as an antioxidant, vitamin C may result in quicker shock resolution in paediatric septic shock.

The Surviving Sepsis Campaign guidelines recommend more research on vitamin C and that this therapy not be employed outside clinical trials.^6^ The combination of vitamin C and hydrocortisone has been proposed as a promising intervention to enhance recovery from septic shock, based on initial observations,^10^ with a need for well-conducted multicentre, multinational paediatric randomised controlled trials investigating this intervention.

To address this knowledge gap, the “Resuscitation in Paediatric Septic Shock using Vitamin C and Hydrocortisone” (RESPOND) trial investigates whether the administration of intravenous (IV) vitamin C (100 mg/kg 6 h) with hydrocortisone (1 mg/kg 6 h) in children <18 years with septic shock will result in quicker shock resolution compared with hydrocortisone alone and compared with standard care.^11^ Here we describe the RESPOND statistical analysis plan (SAP).

### 1.1 Objectives

The primary objective of the trial is to determine whether vitamin C and hydrocortisone result in increased vasopressor-free survival in comparison to hydrocortisone alone and in comparison to standard care. The secondary objectives of the study are to assess the impact of the use of vitamin C and hydrocortisone on 28-day mortality, survival free of organ support, and paediatric intensive care unit (PICU) length of stay. We will also assess health-related quality of life (HRQoL), functional status, and neurodevelopmental vulnerability at 6 months post-enrolment, and hospitalisation-related costs.

## 2 Methods

### 2.1 Trial design

The RESPOND trial is a multinational, parallel, three-arm randomised controlled trial in infants and children <18 years of age with septic shock requiring inotropes/vasopressors.^11^ The RESPOND trial is registered in the Australian New Zealand Clinical Trials Registry (ACTRN12621000247875) with ethical approval in Australia (HREC/20/QCHQ/69922, dated 21/12/2020), New Zealand (2022 PR 10366, dated 14/06/2021), India (2023-17703, dated 26/04/2023), and Brazil (59010122.8.1001.0076, dated 10/04/2023). This SAP is based on protocol version 1.7 (current approved version; protocol modification history detailed in Supplementary Material S2) and reported in line with published guidance (Supplementary Material S3).^12^

### 2.2 Intervention

A detailed description of the intervention was previously published.^11^ In brief, eligible participants will undergo standard initial septic shock management including fluid resuscitation, intravenous antibiotics, and an inotrope/vasopressor as per institutional practice,^13^ and be randomised in a 1:1:1 allocation to one of three treatment arms: vitamin C and hydrocortisone; hydrocortisone alone; or standard care (no vitamin C, but hydrocortisone only optional at the discretion of the treating physician). Sites in Australia, New Zealand, and Brazil are using a sodium ascorbate formulation manufactured in Australia. Due to drug import restrictions, Indian sites are using a locally sourced product. Study treatments are discontinued after shock resolution—defined as cessation of inotropes/vasopressors for at least 4 h—or after a maximum of 72 h. All other care is provided at the discretion of the treating physician.

### 2.3 Randomisation

Treatment assignment for eligible patients is undertaken using a centralised, web-based randomisation interface (REDCap,^14^^,^^15^ hosted by The University of Queensland) with Australian and New Zealand (ANZ) sites also having access to backup sealed opaque envelopes. The variable block randomisation schedule was developed using computer-generated random numbers, stratified by administration of steroids prior to enrolment and hospital site.

### 2.4 Sample size

A total of 384 participants across all study sites will be recruited.^11^ Preplanned interim analyses to assess pretrial sample size assumptions assessed the primary outcome measure distribution after the recruitment of 150 participants in ANZ and 50 participants in Indian sites. Analysis results were reviewed by the Data and Safety Monitoring Board (DSMB) and Trial Steering Committee, and no changes to the sample size were recommended.

### 2.5 Framework

The final analyses will be conducted using a two-sided superiority hypothesis testing framework. Both interventions (hydrocortisone and vitamin C; hydrocortisone alone) will be compared with standard care.

### 2.6 Interim analyses and stopping guidance

The interim analysis approach for reviewing the appropriateness of pre-trial assumptions is detailed in Section 2.4. Interim analyses assessing safety were conducted after 33, 66, and 100 children were enrolled and have continued every 50 patients. At the time of initial interim safety analyses only ANZ sites were recruiting. Given the difference in study formulation used (see Section 2.2) and potential differences in casemix, similar safety analyses are conducted after 33, 66, and 100 patients have been recruited in each country outside ANZ. Safety data are presented to the DSMB (Supplementary Material S4) at each timepoint using masked group identifiers to maintain blinding. Based on interim safety analyses to date, the DSMB has recommended trial continuation with no amendments. There were no predefined interim efficacy analyses and no formal rules to stop for efficacy or futility. Therefore, the final significance level remains at 5%.

### 2.7 Timing of final analysis

Final analyses will occur in two stages: firstly, analysis of short-term outcomes (primary analysis), and secondly, analysis of long-term outcomes (Supplementary Table S1). The primary analysis will occur following completion of data monitoring of all screening, intervention, and short-term outcome data and the associated database lock (Supplementary Material S5). Analysis of long-term outcomes will be undertaken after completion of data monitoring for these outcomes. The RESPOND SAP was finalised prior to recruitment of the last patient and prior to locking the database.

### 2.8 Timing of outcome assessments

The primary outcome will be assessed at seven days post-randomisation (Supplementary Table S1). The secondary outcome of alive and free of multiple organ dysfunction will be assessed at 72 h postrandomisation. Secondary outcomes including survival free of organ support, PICU-free survival, length of stay, and mortality will be assessed at 28 days post-randomisation. Long-term outcomes including HRQoL, functional status, and neurodevelopmental vulnerability, and hospitalisation-related costs will be assessed at six months postrandomisation.

## 3 Statistical principles

### 3.1 Confidence intervals and p-values

Statistical significance will be set at the 0.05 level. Analysis of the primary outcome measure will be reported with p-values; however, 95% confidence intervals (CIs) will be the primary mode of presentation for all other analyses. The family-wise error rate will be controlled using a Dunnett simultaneous testing procedure.

### 3.2 Adherence and protocol deviations

Protocol deviations relate to several trial aspects, including consenting, randomisation, intervention delivery, and long-term follow-up. Adherence to the intervention is defined as the participant receiving all study medications as prescribed by the protocol. Protocol deviations are captured for each participant (Supplementary Material S6) and confirmed during data monitoring (Supplementary Material S5). All protocol deviations will be presented as supplementary material in the results manuscript, and, where relevant, presented by randomised treatment group. Only deviations related to intervention delivery will be used to ascertain adherence to the intervention.

### 3.3 Analysis populations

The intention-to-treat principle (i.e. patients who were randomised, consented, and did not withdraw consent for data use) will be applied to define the analysis population for the primary analysis. As such, each enrolled patient will be analysed based on the allocated treatment group, independent of compliance with the treatment delivered. Additionally, a modified per-protocol analysis will be undertaken and reported. For the modified per-protocol analysis population, where a study therapy (hydrocortisone and/or vitamin C) was commenced (regardless of whether it was the therapy that was randomised), the patient will be allocated to the treatment group aligned with the treatment actually received. If no study therapy (hydrocortisone and/or vitamin C) was received, an enrolled patient will be allocated to the standard care group. Long-term outcomes will be analysed using the same principles but presented separately to the short-term outcomes.

## 4 Trial population

### 4.1 Screening data

Infants and children who met inclusion criteria will be reported as screened, with reasons for subsequent exclusion listed.

### 4.2 Eligibility

Any infants and children with a clinical diagnosis of septic shock, receiving inotropes/vasopressors, and admitted to one of the participating PICUs are eligible for inclusion. Specific inclusion and exclusion criteria are detailed previously.^11^

### 4.3 Recruitment

**Fig. 1:**
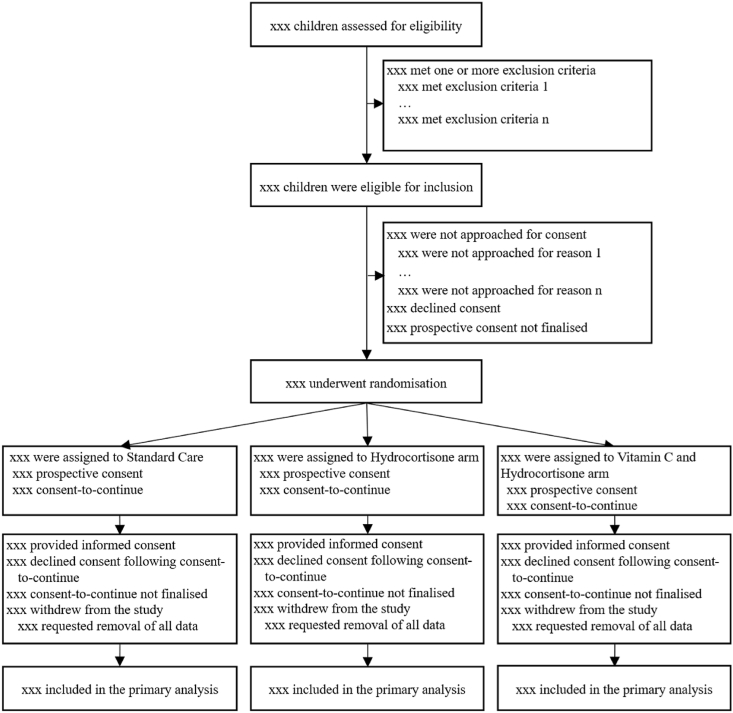
Proposed participant flow diagram.

The flow of patients through the trial will be presented graphically, based on the Consolidated Standards of Reporting Trials (CONSORT)^16^(Fig. 1). This will describe screened patients, those meeting exclusion criteria, eligible patients, the consent process (including a clear description of those randomised under prospective consent and consent-to-continue, as per our previous trials^8^^,^^17^), randomisation into each of the study arms, with the documentation of the primary outcome. When reporting the long-term outcomes, we will present an extended CONSORT to include follow-up status (Supplementary Figure S1). Participant recruitment over the course of the study will be reported graphically, along with the start and stop dates of the trial as well as when each site commenced.

### 4.4 Withdrawal/follow-up

For participants who consented, and subsequently withdraw consent, data will be excluded from the analyses, unless permission is granted to use data recorded prior to the point of consent withdrawal. Patients who are enrolled under the consent-to-continue model (i.e. study treatments are administered prior to obtaining consent) and subsequently decline consent, will be treated as a decline, i.e. the patient's data will not be used for analyses. Such instances will be reported in the participant flow diagram (Fig. 1). As there is an expected attrition with respect to completion of long-term outcome assessments, characteristics of participants who were lost to follow-up will be presented alongside those who completed follow-up.

### 4.5 Baseline patient characteristics

Baseline characteristics at the time of randomisation will be reported using frequencies and percentages (categorial variables) and mean (standard deviation) or median (interquartile range) for continuous variables (dependent on variable distribution) for each of the three treatment groups (Table 1). Statistical comparison between groups will not be undertaken.

**Table 1:** Baseline characteristics of participants enrolled in the RESPOND trial.

Characteristic	Vitamin C and hydrocortisone N=	Hydrocortisone only N=	Standard care N=
**Age at randomisation (years)***mean (SD)/median (IQR)*			
**Weight (kg)***mean (SD)/median (IQR)*			
**Female sex***n (%)*			
**Country of recruitment***n (%)*			
Australia			
Brazil			
India			
New Zealand			
**Ethnicity***n (%)*			
Caucasian			
Aboriginal/Torres Strait Islander			
Asian			
African			
Indian			
Middle Eastern			
Māori			
Pacific Islander			
Pardo			
Mixed or other			
Not disclosed			
Unknown			
**Primary reason(s) for presentation to hospital**^a^			
Central nervous system disease *n (%)*			
Elective surgical procedure *n (%)*			
Endocrinology (including diabetes) *n (%)*			
Intoxication *n (%)*			
Gastrointestinal *n (%)*			
Heart disease (congenital/acquired) *n (%)*			
Malignancy *n (%)*			
Metabolic *n (%)*			
Renal *n (%)*			
Respiratory disease *n (%)*			
Rheumatology *n (%)*			
Sepsis/infection *n (%)*			
Trauma/burns *n (%)*			
Other *n (%)*			
**Comorbidities***n (%)*			
Any comorbidity *n (%)*			
Congenital or genetic defect *n (%)*			
Chronic respiratory disorders *n (%)*			
Chronic neurological disorders *n (%)*			
Prematurity *n (%)*			
Oncology/malignancy *n (%)*			
Gastrointestinal *n (%)*			
Metabolic *n (%)*			
Technology dependency *n (%)*			
Cardiovascular *n (%)*			
Renal/urologic *n (%)*			
Haematologic/immunologic *n (%)*			
Mental health/behavioural *n (%)*			
Other *n (%)*			
**ICU admission source***n (%)*			
Operating theatre/recovery			
Operating theatre (direct admission from another ICU/NICU via operating theatre)			
Emergency department			
Hospital ward			
Direct ICU admission			
Other ICU/NICU same hospital			
**Baseline mPOPC***mean (SD)/median (IQR)*			
**Baseline functional status score***mean (SD)/median (IQR)*			
**Baseline health-related quality of life**^b^*mean (SD)/median (IQR)*			
**Observations at randomisation**			
Heart rate *mean (SD)/median (IQR)*			
Respiratory rate *mean (SD)/median (IQR)*			
Systolic blood pressure *mean (SD)/median (IQR)*			
Mean blood pressure *mean (SD)/median (IQR)*			
Diastolic blood pressure *mean (SD)/median (IQR)*			
Temperature *mean (SD)/median (IQR)*			
SpO_2_*mean (SD)/median (IQR)*			
FiO_2_ at time of SpO2 *mean (SD)/median (IQR)*			
PaO_2_/FiO_2_ ratio *mean (SD)/median (IQR)*			
Capillary refill time			
Not measured *n (%)*			
<2 s *n (%)*			
2-5 s *n (%)*			
>5 s *n (%)*			
Glasgow coma score *mean (SD)/median (IQR)*			
Dilated unresponsive pupils *n (%)*			
**Laboratory**			
pH *mean (SD)/median (IQR)*			
Base excess [mmol/l] *mean (SD)/median (IQR)*			
PaO_2_ [mmHg] *mean (SD)/median (IQR)*			
pCO_2_ [mmHg] *mean (SD)/median (IQR))*			
Lactate [mmol/l] *mean (SD)/median (IQR)*			
Glucose [mmol/l] *mean (SD)/median (IQR)*			
Sodium [mmol/l] *mean (SD)/median (IQR)*			
Chloride [mmol/l] *mean (SD)/median (IQR)*			
Creatinine [μmol/l] *mean (SD)/median (IQR)*			
Bilirubin [μmol/l] *mean (SD)/median (IQR)*)			
Alanine aminotransferase level [U/L] *mean (SD)/median (IQR)*			
International normalized ratio *mean (SD)/median (IQR)*			
Platelets^c^ [x10^9^/L] *mean (SD)/median (IQR)*			
White cell count^c^ [x10^9^/L *mean (SD)/median (IQR)*			
Absolute neutrophil count^c^ [x10^9^/L] *mean (SD)/median (IQR)*			
Haemoglobin [g/L] *mean (SD)/median (IQR)*			
C-reactive protein [mg/L] *mean (SD)/median (IQR)*			
**Organ dysfunction at randomisation**			
pSOFA *mean (SD)/median (IQR)*			
Phoenix sepsis score *mean (SD)/median (IQR)*			
**Treatment at or prior to randomisation**			
IV steroids given to treat septic shock in the 24 h before randomisation^c^*n (%)*			
Vasoactive inotrope score *mean (SD)/median (IQR)*			
Respiratory support *n (%)*			
None			
Standard oxygen therapy			
High flow oxygen therapy			
Noninvasive ventilation			
Invasive ventilation			
ECMO *n (%)*			
Renal replacement therapy *n (%)*			
Total amount of IV fluid boluses administered in the last 4 h prior to randomisation (mL/kg) *mean (SD)/median (IQR)*			

ECMO, extracorporeal membrane oxygenation; ICU, intensive care unit; IQR, interquartile range; IV, intravenous; pSOFA, paediatric Sequential Organ Failure Assessment; mPOPC, modified Paediatric Overall Performance Category; NICU, neonatal intensive care unit; SD, standard deviation.

a Multiple responses can be chosen.

b Health-related quality of life measured using the Paediatric Quality of Life Inventory Total Score.

c Used for stratification.

## 5 Analysis

### 5.1 Outcome definitions

The primary outcome is defined as duration free of inotropes/vasopressors, censored at seven days post-randomisation; patients dying within seven days of randomisation will be recorded as zero (Supplementary Table S1). Given the open-label pragmatic trial design, and that weaning of vasoactive agents is not protocolised, the primary outcome is potentially subject to bias. The risk of bias is expected to be mitigated by the robust trial methodology and protocol fidelity. Despite the lack of blinding, it is unlikely that clinicians substantially altered their usual vasoactive weaning practices based on treatment allocation. Secondary outcomes are defined in Supplementary Table S1.

### 5.2 Analysis methods

#### 5.2.1 Primary outcome

The primary outcome will be analysed using a single quantile regression model, comparing the two intervention groups with standard care. Stratification variables (use of steroids preenrolment and site) will be included as fixed effects in the model. If >10% of patients have been enrolled into the RESPOND trial more than once, patient will also be included in the model as a random effect. An unadjusted model will also be reported. Estimates of difference, 95% CIs, and p-values for the adjusted model will be reported (Table 2). Adjusted p-values and simultaneous confidence intervals will be obtained using Dunnett's many-to-one procedure applied to the joint Wald statistics from the quantile regression model and reported. Assumptions of the models will be tested and reported on. The primary analysis code and results will be independently verified by a statistician independent of the trial.

**Table 2:** Primary outcomes in the total trial cohort and subgroups as per intention-to-treat analysis.

Outcome: Vasopressor free days Time alive and free of inotropes/vasopressors, censored at 7 days (168 h) postrandomisation	Mean (SD)/median (IQR)				Adjusted estimate of difference (95% CI)^a^		p-value	
	Vitamin C and hydrocortisone N=	Hydrocortisone only N=	Standard care N=		Vitamin C and hydrocortisone vs standard care	Hydrocortisone only vs standard care	Vitamin C and hydrocortisone vs standard care	Hydrocortisone only vs standard care
**Total trial cohort**								
**Subgroup: Intravenous steroids given to treat septic shock in the 24 h before randomisation (stratification variable)**^c^**^,^**^b^								
No								
Yes								
**Subgroup: Organ dysfunction at randomisation**^c^								
pSOFA <2								
pSOFA ≥2								
**Subgroup: Patient age**^c^								
<1 year of age								
1-<5 years of age								
>5 years of age								
**Subgroup: Comorbidity**^c^								
No								
Yes								
**Subgroup: Origin of infection**^c^								
Community-acquired								
Hospital-acquired								
**Subgroup: Vitamin C formulation**^c^								
Sodium ascorbate								
Ascorbic acid								

IQR, interquartile range; CI, confidence interval; pSOFA, paediatric Sequential Organ Failure Assessment *(Matics TJ, Sanchez-Pinto LN. Adaptation and validation of a pediatric sequential organ failure assessment score and evaluation of the sepsis-3 definitions in critically ill children. JAMA Pediatrics. 2017 Oct 1;171(10):e172352)*.

a Adjusted for stratification variables (steroid use prior to randomisation, site).

b Adjusted for stratification variable (site).

c p-value represents interaction term.

#### 5.2.2 Secondary outcomes

Binary outcome measures (e.g. mortality) will be analysed using a logistic regression model, adjusting for treatment group and administration of steroids prior to enrolment as fixed effects, and hospital site as a random effect (Table 3). The same principles will be followed for continuous outcomes (both short- and long-term); generalised linear models with adjustment for treatment group and stratification variable as fixed effects and site as a random effect, with reporting of mean differences (unadjusted and adjusted) and 95% CIs. If necessary, quantile regression will replace linear regression in the instance of highly skewed data. Length of stay outcomes will be visually presented using a Kaplan–Meier plot; a Cox proportional hazard model will be used to assess differences between treatment groups, with treatment group and stratification variables as fixed effects and site as a random effect (i.e., utilising a shared frailty model), with discharge considered the event and censoring at time of death (i.e. using a cause-specific approach), with the hazard ratio and 95% CI presented as an estimate of treatment effect. The proportionality assumption will be inspected visually and reported. Key assumptions of all models will be tested and reported on.

**Table 3:** Secondary short-term outcomes in the intention-to-treat trial cohort.

Outcome	Mean (SD)/median (IQR)/n (%)			Adjusted estimate of difference (95% CI)^a^	
	Vitamin C and hydrocortisone N=	Hydrocortisone only N=	Standard care N=	Vitamin C and hydrocortisone vs standard care	Hydrocortisone only vs standard care
Alive and free of multiorgan dysfunction^b^*n (%)*					
PICU-free survival *mean (SD)/median (IQR)*					
Survival free of organ support *mean (SD)/median (IQR)*					
Survival free of cardiovascular support *mean (SD)/median (IQR)*					
Survival free of ventilation *mean (SD)/median (IQR)*					
28-day mortality *n (%)*					
Length of stay in PICU *mean (SD)/median (IQR)*					
Length of stay in hospital *mean (SD)/median (IQR)*					
Adverse events					
Hyperglycaemia *n (%)*					
Hypoglycaemia *n (%)*					
Hypernaetremia *n (%)*					
Hospital-acquired microbiologically confirmed infection *n (%)*					
Oxalate nephropathy *n (%)*					
Haemolysis *n (%)*					
Worsening of liver function *n (%)*					

SD, standard deviation; IQR, interquartile range; CI, confidence interval; PICU, paediatric intensive care unit.

a Adjusted for stratification variables (steroid use prior to randomisation, site).

b Multiorgan dysfunction is defined as pSOFA (paediatric Sequential Organ Failure Assessment) organ-specific subscore increase in at least 2 organs from randomisation to 72 h post-randomisation.

We will undertake several *a priori*-defined subgroup analyses, examining the primary outcome only:

• Administration of steroids prior to enrolment (stratification variable);
• Severity upon presentation as per organ dysfunction scores (baseline pSOFA^18^ ≥2, regarding Phoenix Sepsis Score see comment below);
• Age (<1 year of age, 1 to <5 years of age, ≥5 years of age);
• Children with comorbidity (haematologic, immunologic, oncology);
• Origin of infection (community-, hospital-acquired infection^19^); and
• Vitamin C formulation (sodium ascorbate vs ascorbic acid; secondary outcomes will also be reported for this subgroup in the supplement).

Subgroup analyses will compare both interventions groups to standard care, using the methods described above with the addition of the subgroup variable and its related interaction term. The treatment effect and 95% CI within each subgroup (without a p-value) and only the p-value associated with the interaction term (between the subgroup variable and treatment group), will be reported, alongside the descriptive statistics for the outcome under investigation (Table 2). Graphical representation of the results will be displayed through a forest plot.

As the trial started before the Phoenix Sepsis Criteria^3^ became available, a sensitivity analysis restricted to children meeting the Phoenix Sepsis Criteria for septic shock at enrolment will be performed. Additionally, a sensitivity analysis will be undertaken for the HRQoL outcome, incorporating baseline HRQoL as a covariate.

### 5.3 Missing data

Based on our previous trials,^8^^,^^17^ we anticipate a very low rate of missing data for the primary outcome. However, if there are >10% missing data for the primary outcome, multiple imputation will be performed and reported as a sensitivity analysis. Based on our previous work, it is anticipated that there will be substantial missing data for long-term outcomes. As such, a sensitivity analysis will be undertaken for the primary long-term outcome (health-related quality of life at six months postrandomisation; Table S1) using multiple imputation via the chained equation method, using a similar approach to that used in our previous work.^20^

### 5.4 Additional analyses

In addition to the primary comparisons (i.e. each intervention group versus standard care), three further exploratory analyses will be undertaken for the primary outcome measure:

• the two intervention groups combined (hydrocortisone and vitamin C, hydrocortisone alone) compared with standard care,
• hydrocortisone and vitamin C group compared with the hydrocortisone and standard care groups combined, and
• hydrocortisone and vitamin C compared with hydrocortisone alone,

Additional analyses may be undertaken at the discretion of the Trial Steering Committee, under the advisement of the DSMB, or at the request of journal editors and/or reviewers. Such analyses will be listed as exploratory and declared as post-hoc analyses.

### 5.5 Health economic analyses

We will conduct a within-trial cost-utility analysis (six month horizon) from the health system perspective, comparing each intervention with standard care, restricted to Australian sites to ensure consistent costing.

Health-related quality of life at 6 months will be measured using the Paediatric Quality of Life Inventory (PedsQL). Utility weights will be derived using the published mapping algorithm^21^ to convert to CHU9D-based health-state utilities, and combined with survival data to generate patient-level quality-adjusted life years (QALYs). Sensitivity analyses will test alternative assumptions regarding utility values for PICU stay and recovery trajectories.

Resource use will be captured from case report forms, hospital administrative data, and 6-month follow-up questionnaires. Costs will include direct hospitalisation costs and intervention costs, standardised to 2025 Australian dollars without discounting.

Incremental costs, QALYs, and net monetary benefit will be estimated using regression models adjusted for baseline prognostic factors and accounting for site clustering, with uncertainty quantified by nonparametric bootstrapping to generate 95% CIs, cost-effectiveness planes, and acceptability curves across willingness-to-pay thresholds of A$30,000–A$100,000 per QALY. Long-term cost-effectiveness will be explored using a Markov model informed by published data.

### 5.6 Harms

All adverse events (AEs) defined in the protocol will be reported per treatment group,^11^ and the type, grade, and relatedness of all AEs will be summarised. Independent medical monitors are reviewing all AEs to verify the investigator's assessment of relatedness. Safety outcomes, as per Section 5.1, will be compared between treatment groups as per the analysis approach in Section 5.2.2.

### 5.7 Statistical software

The statistical analysis will be undertaken using StataSE version 17 (StataCorp Pty Ltd, College Station, Texas), or the most recent version available at the time of analysis.

## CRediT authorship contribution statement

The statistical analysis plan first draft was designed by KSG, SR, RL, and LJS. KG and RL designed and drafted all statistical analyses and outputs contained in this manuscript. SR was responsible for all operational details, clinical interpretation, and procedural study components. Review, comments, and feedback was provided by all other authors. All authors attest to having approved the final manuscript.

## Funding

This work is supported by funding from the National Health and Medical Research Council (Australia; GNT2014937), the Financial Markets for Children Foundation (Australia; 2019-226), the Children's Hospital Foundation, (Brisbane, Australia; 50282) and the Wolfermann-Nägeli-Stiftung (Switzerland). Biobanking is supported by funding from the Medical Research Futures Fund (Australia; GHFMPACI000004). SR is supported by a Children's Hospital Foundation Early Career Fellowship (Australia). KSG are supported by the National Health and Medical Research Council Investigator Grants (Australia). LJS is supported by the Thomas & Doris Ammann Foundation and the NOMIS Foundation (Switzerland). The vitamin C used in Australia, New Zealand, and Brazil is provided free of cost by Biological Therapies, Australia. The funding sources have no involvement in study design, nor will have input into analyses, or interpretation or reporting of the results.

## Conflict of interest

The authors declare the following financial interests which may be considered as conflict of interests: Kristen Gibbons reports vitamin C was provided by Biological Therapies. Kristen Gibbons reports financial support was provided by the National Health and Medical Research Council. Luregn Schlapbach reports financial support was provided by the Financial Markets for Children Foundation and Wolfermann–Nägeli-Stiftung Foundation. Sainath Raman reports financial support was provided by the Children's Hospital Foundation. Other authors declare that they have no known competing financial interests or personal relationships that could have influenced the work reported in this paper.
