# Supplementary material for: Resuscitation in paediatric septic shock using vitamin C and hydrocortisone (RESPOND): The RESPOND randomised controlled trial statistical analysis plan
Source: Crit Care Resusc. 2026 Jun 16;28(3):100183. doi: 10.1016/j.ccrj.2026.100183 (PMC13284511; doi:10.1016/j.ccrj.2026.100183)
Supplement: Multimedia component 1 [file mmc1.docx]

**Resuscitation in paediatric septic shock using vitamin C and hydrocortisone (RESPOND): The RESPOND randomised controlled trial statistical analysis plan**

Kristen S. Gibbons, Sainath Raman, Lalitha AV, Marino Festa, Shane George, Renate Le Marsney, Paula Lister, Debbie A. Long, Karthi Nallasamy, Anton Pak, Daniela Carla de Souza, Barbara Zangerl, Luregn J. Schlapbach on behalf of the RESPOND Study Group and the Australian and New Zealand Intensive Care Society Paediatric Study Group (ANZICS PSG)

**Corresponding Author:**

Professor Kristen Gibbons

Children’s Intensive Care Research Program

Child Health Research Centre

The University of Queensland

Brisbane, QLD, Australia.

Email: [k.gibbons@uq.edu.au](mailto:k.gibbons@uq.edu.au)

**Supplemental Digital Content**

**S1.** Resuscitation in Paediatric Septic Shock using Vitamin C and Hydrocortisone (RESPOND) Study Group

**S2.** List of approved protocol modifications

**S3.** Checklist of Recommended Items to Address in a Clinical Trial Statistical Analysis Plan

**S4.** Data and Safety Monitoring Board Charter for the RESPOND trial

**S5.** Description of data sources and data monitoring plan for the RESPOND trial

**S6.** Listing of protocol deviations recorded for the RESPOND trial

**Figures**

**Figure S1.** Proposed participant flow diagram including long-term outcomes

**Tables**

**Table S1.** Definitions of trial outcomes

**S1.** **Resuscitation in Paediatric Septic Shock using Vitamin C and Hydrocortisone (RESPOND) Study Group**

Dr Ebor James Jacob, Christian Medical College, Vellore, India; A/Prof Shane George, Mr Kieran Owen, Gold Coast University Hospital, Southport, Australia; Dr Daniela Carla De Souza, Hospital Universitário da Universidade de São Paulo, São Paulo, Brazil; Dr Simon Erickson, Dr Nick Williams, Ms Arielle Jolly, Perth Children’s Hospital, Perth, Australia; Prof Muralidharan Jayashree, Dr Karthi Nallasamy, Post Graduate Institute of Medical Education and Research, Chandigarh, India; Dr Sainath Raman, Ms Michele Cree, Ms Kerry Johnson, Louise Sparkes, Dr Barbara Zangerl, Queensland Children’s Hospital, Brisbane, Australia; Prof Debbie Long, Queensland University of Technology, Brisbane, Australia; Dr Puneet Singh, Ms Claire Collins, Sydney Children’s Hospital, Sydney, Australia; Dr Lalitha AV, St John’s Hospital, Bangalore, India; Dr David Buckley, Dr John Beca, Ms Claire Sherring, Starship Children’s Hospital, Auckland, New Zealand; Dr Paula Lister, Ms Charlotte Moore, Sunshine Coast University Hospital, Sunshine Coast, Australia; Dr Marino Festa, Ms Heidi Baillie, The Children’s Hospital at Westmead, Sydney, Australia; Prof Bala Venkatesh, Prof Vivekanand Jha, Abhinav Bassi, Nikita Bathla, A/Prof Naomi Hammond, The George Institute, New Delhi, India, and Sydney, Australia; Prof Kristen Gibbons, Ms Renate Le Marsney, Ms Trang Pham, Prof Luregn J Schlapbach, The University of Queensland, Brisbane, Australia, and University Children’s Hospital Zurich, Zurich, Switzerland.

The RESPOND Study Group acknowledges the contribution of late Professor Rinaldo Bellomo, Melbourne, to the design and conduct of this trial.

**S2. List of approved protocol modifications**

| **Version Number and Date** | **Ethics Committee Approval Date** | **List of Modifications** |
| --- | --- | --- |
| *1.0* | *-* | *Original submission* |
| 1.1 | 11/12/2020 | - Process to follow in case of enrolment of oncology patients - Time frame for obtaining consent to continue must not exceed 24 hours (previously 72 hours). |
| 1.2 | 04/03/2021 | - Study exclusion criteria updated to remove “Patients with known or suspected scurvy” - Research Coordinator changed - New RESPOND generic email address - Randomisation updated to reflect new computer-based randomisation process - Small grammatical errors corrected throughout |
| 1.3 | 06/05/2021 | - ANZCTR number added - Additional secondary outcomes added: (1) Neurodevelopmental vulnerability at 6 months post sepsis, (2) new onset AKI - Randomisation process updated, with new screenshot included to outline the electronic based method |
| 1.4 | 01/06/2021 | - Chronic renal failure removed from exclusion criteria - Addition of clinical staff to the randomisation process - Site investigator list updated - Study management committee updated |
| 1.5 | 17/09/2021 | - Primary outcome changed to time alive free of vasopressors censored at 7 days - Thiamine removed from the intervention - PedsQL added to baseline observations. - Consumer representative added - Study sites in Brazil added |
| 1.6 | 01/09/2022 | - Additional investigator added - Amended Figure 3 on page 25 – study procedures - Investigators title change - Additional safety features and interim analysis plan - Added exclusion medications - cyanocobalamin or Deferoxamine for their co-morbid conditions - Included Sponsor/Updated Funding and duration of trial - Updated current literature - Amended follow up process - Amended DSMB Roles and Responsibilities |
| 1.7 | 08/11/2024 | - Update to Trial Steering Committee composition - Inclusion of Indian sites and investigators - Amended timeframes hospital-acquired microbiologically confirmed infection - Updated long-term follow-up processes |

ANZCTR Australian New Zealand Clincial Trials Registry; AKI acute kidney injury; PedsQL Pediatric Quality of Life Inventory; DSMB Data Safety Monitoring Board

**S3.** **Checklist of Recommended Items to Address in a Clinical Trial Statistical Analysis Plan^*^**

| **Section/Item** | **Index** | **Description** | **Reported on Page #** |
| --- | --- | --- | --- |
| *Section 1: Administrative Information* | | | |
| Title and trial registration | 1a | Descriptive title that matches the protocol, with SAP either as a forerunner or subtitle, and trial acronym (if applicable) | 1 |
|  | 1b | Trial registration number | 3 |
| SAP version | 2 | SAP version number with dates | N/A |
| Protocol version | 3 | Reference to version of protocol being used | 5 |
| SAP revisions | 4a | SAP revision history | N/A – nil revisions |
|  | 4b | Justification for each SAP revision |  |
|  | 4c | Timing of SAP revisions in relation to interim analyses, etc |  |
| Roles and responsibility | 5 | Names, affiliations, and roles of SAP contributors | 16 |
| Signatures of: | 6a | Person writing the SAP | N/A |
|  | 6b | Senior statistician responsible |  |
|  | 6c | Chief investigator/clinical lead |  |
| *Section 2: Introduction* | | | |
| Background and rationale | 7 | Synopsis of trial background and rationale including a brief description of research question and brief justification for undertaking the trial | 4 |
| Objectives | 8 | Description of specific objectives or hypotheses | 5 |
| *Section 3: Study Methods* | | | |
| Trial design | 9 | Brief description of trial design including type of trial (e.g., parallel group, multi-arm, crossover, factorial) and allocation ratio and may include brief description of interventions | 5 |
| Randomisation | 10 | Randomization details, e.g., whether any minimization or stratification occurred (including stratifying factors used or the location of that information if it is not held within the SAP) | 6 |
| Sample size | 11 | Full sample size calculation or reference to sample size calculation in protocol (instead of replication in SAP) | 6 |
| Framework | 12 | Superiority, equivalence, or noninferiority hypothesis testing framework, including which comparisons will be presented on this basis | 7 |
| Statistical interim analyses and stopping guidance | 13a | Information on interim analyses specifying what interim analyses will be carried out and listing of time points | 7 |
|  | 13b | Any planned adjustment of the significance level due to interim analysis | 7 |
|  | 13c | Details of guidelines for stopping the trial early | 7 |
| Timing of final analysis | 14 | Timing of final analysis, e.g., all outcomes analysed collectively or timing stratified by planned length of follow-up | 7 |
| Timing of outcome assessments | 15 | Time points at which the outcomes are measured including visit “windows” | 8 |
| *Section 4: Statistical Principles* | | | |
| Confidence intervals and *P* values | 16 | Level of statistical significance | 8 |
|  | 17 | Description and rationale for any adjustment for multiplicity and, if so, detailing how the type 1 error is to be controlled | 8 |
|  | 18 | Confidence intervals to be reported | 8 |
| Adherence and protocol deviations | 19a | Definition of adherence to the intervention and how this is assessed including extent of exposure | 8 |
|  | 19b | Description of how adherence to the intervention will be presented | 8 |
|  | 19c | Definition of protocol deviations for the trial | 8; Supplementary Material S6 |
|  | 19d | Description of which protocol deviations will be summarized | Supplementary Material S6 |
| Analysis populations | 20 | Definition of analysis populations, e.g., intention to treat, per protocol, complete case, safety | 9 |
| *Section 5: Trial Population* | | | |
| Screening data | 21 | Reporting of screening data (if collected) to describe representativeness of trial sample | 9 |
| Eligibility | 22 | Summary of eligibility criteria | 9 |
| Recruitment | 23 | Information to be included in the CONSORT flow diagram | 10 |
| Withdrawal/follow-up | 24a | Level of withdrawal, e.g., from intervention and/or from follow-up | 10 |
|  | 24b | Timing of withdrawal/lost to follow-up data | 10 |
|  | 24c | Reasons and details of how withdrawal/lost to follow-up data will be presented | 10 |
| Baseline patient characteristics | 25a | List of baseline characteristics to be summarized | Table 1 |
|  | 25b | Details of how baseline characteristics will be descriptively summarized | 10 |
| *Section 6: Analysis* | | | |
| Outcome definitions |  | List and describe each primary and secondary outcome including details of: | Supplementary Table S1 |
|  | 26a | specification of outcomes and timings. If applicable include the order of importance of primary or key secondary end points (e.g., order in which they will be tested) | Supplementary Table S1 |
|  | 26b | specific measurement and units (e.g., glucose control, hbA1c [mmol/mol or %]) | Supplementary Table S1 |
|  | 26c | any calculation or transformation used to derive the outcome (e.g., change from baseline, QoL score, time to event, logarithm, etc) | Supplementary Table S1 |
| Analysis methods | 27a | what analysis method will be used and how the treatment effects will be presented | 11-13 |
|  | 27b | any adjustment for covariates | 11-12 |
|  | 27c | methods used for assumptions to be checked for statistical methods | 12 |
|  | 27d | details of alternative methods to be used if distributional assumptions do not hold, e.g., normality, proportional hazards, etc | 12 |
|  | 27e | any planned sensitivity analyses for each outcome where applicable | 13 |
|  | 27f | any planned subgroup analyses for each outcome including how subgroups are defined | 12 |
| Missing data | 28 | Reporting and assumptions/statistical methods to handle missing data (e.g., multiple imputation) | 13 |
| Additional analyses | 29 | Details of any additional statistical analyses required, e.g., complier-average causal effect10 analysis | 13 |
| Harms | 30 | Sufficient detail on summarising safety data, e.g., information on severity, expectedness, and causality; details of how adverse events are coded or categorized; how adverse event data will be analysed, i.e., grade 3/4 only, incidence case analysis, intervention emergent analysis | 15 |
| Statistical software | 31 | Details of statistical packages to be used to carry out analyses | 15 |
| References | 32a | References to be provided for nonstandard statistical methods | N/A |
|  | 32b | Reference to Data Management Plan | Supplementary Material S6 |
|  | 32c | Reference to the Trial Master File and Statistical Master File | N/A |
|  | 32d | Reference to other standard operating procedures or documents to be adhered to | N/A |

CONSORT, Consolidated Standards of Reporting Trials; hbA_1c_, haemoglobin A_1c_; QoL, quality of life; SAP, statistical analysis plan

* Gamble C, Krishan A, Stocken D, Lewis S, Juszczak E, Doré C, Williamson PR, Altman DG, Montgomery A, Lim P, Berlin J. Guidelines for the content of statistical analysis plans in clinical trials. *JAMA.* 2017 Dec 19;318(23):2337-43.

**S4. Data and Safety Monitoring Board Charter for the RESPOND trial**

An independent Data and Safety Monitoring Board (DSMB) has been convened to assess the progress of the Resuscitation in Paediatric Septic Shock Using Vitamin C and Hydrocortisone (RESPOND) study, as well as review accruing safety data, and provide recommendations to the RESPOND study team. The members of the DSMB serve in an individual capacity and provide their expertise and recommendations. The DSMB will review cumulative study data to evaluate safety, study conduct, and scientific validity and data integrity of the study. This Charter outlines the roles and responsibilities and serve as the Standard Operating Procedure (SOP) for the DSMB.

**COMPOSITION OF THE DSMB**

The Board will be composed of four members (inclusive of the DSMB Chair). The DSMB includes experts in, or representatives of, the fields of intensive care medicine, clinical trials, and statistics.

Quorum: A quorum will occur when one statistician and one clinician, including the Chair (unless otherwise agreed), occurs. Without a quorum a meeting will not be held, unless alternate arrangements have been made by the Chair in agreement with RESPOND Study Management Team, where documents can be reviewed remotely and written review comments will be provided to the Chair. A separate electronic or documented review of materials may also be requested of members in certain circumstances.

Each member will be appointed for the length of the RESPOND study.

**INDEPENDENCE OF THE DSMB**

It is essential that the judgment of members of the DSMB not be influenced by factors other than those necessary to maintain subject safety and to preserve the integrity of the study. Persons who have an apparent financial, intellectual, or other interests should not be a DSMB participant for the evaluation of that product. Independence is essential to ensure that DSMB members are objective and capable of an unbiased assessment of the study's safety and efficacy data. The following will ensure the independence of the DSMB:

- DSMB members will not be supervised by any study investigator of a protocol currently under review by this DSMB or participate as investigators in any study currently under review by this DSMB.
- Members of the DSMB must not have a direct interest in knowing or influencing trial outcome or have a financial or intellectual interest in the outcome of any studies under review.
- DSMB members must disclose all pharmaceutical companies, biotechnology companies, and Clinical Research Organisations (CROs) in which they hold financial interest. Members must disclose all consultancies (direct or indirect) with pharmaceutical companies, biotechnology companies, and CROs.
- Members who have served initially on protocol review teams may participate in the open sessions of the DSMB meeting when that protocol is under review. However, they will be excused from the closed sessions reviewing that protocol.

The RESPOND Coordinating Principal Investigator will be responsible for deciding whether consultancies or the disclosed interests of the members materially affect their objectivity. Members of the DSMB will be responsible for notifying the DSMB Chair and RESPOND Study Management Team of any changes of interest in pharmaceutical companies, biotechnology companies, or CROs, including consultancies. In such cases, the DSMB meeting minutes will document the disclosure of the potential conflict of interest and the outcome of the discussion (e.g., abstention of member from voting, recusal from discussion). The RESPOND Coordinating Principal Investigator will decide whether any of these relationships results in a conflict of interest which would preclude involvement on the DSMB. Members of the DSMB who develop potential or significant perceived conflicts of interest will be asked to resign from the DSMB. Members will be polled at the beginning of each DSMB meeting to disclose whether status has changed.

**RESPONSIBILITIES OF THE DSMB**

As this DSMB is constituted for a single protocol, DSMB members should only agree to serve if they are generally supportive of the study’s overall aims and general design. This is because the study has already been through a scientific review. The DSMB will consider study-specific data as well as current relevant background knowledge about the disease, test agent, equipment or patient population under study.

Objectives

The primary objective of the DSMB is to monitor the safety of the intervention and the validity and integrity of the data from the RESPOND trial. Additionally, the DSMB will evaluate the pace of recruitment and will make recommendations to the RESPOND Study Management Team regarding the continuation, modification, or termination of the study.

General Responsibilities

The general responsibilities of the DSMB are:

- To evaluate, on an ongoing basis, the accumulating safety assessments to ensure the ongoing safety of study subjects;
- To consider factors external to the study when relevant information becomes available, such as scientific or therapeutic developments that may have an impact on the safety of the participants or the ethics of the study;
- To review all documents upon notification to the DSMB;
- To review the conduct of the study, including protocol violations;
- To review data on participant recruitment, accrual, and retention, as well as assessments of data quality, completeness, and timeliness;
- To protect the confidentiality of the study data and the DSMB discussions; and
- To make recommendations to continue, modify, or terminate the study.

DSMB members will have the ability to review unmasked clinical data. Unmasked clinical data will be discussed only during the closed session of the meeting when only DSMB members are present

**DSMB CHAIR RESPONSIBILITIES**

The following responsibilities are those of the DSMB Chair:

- Serves as a voting member;
- Facilitates the meetings, assists in the development of the agenda, and ensures that the meeting minutes and recommendation(s) are appropriately documented;
- Serves as the primary contact person for the DSMB;
- Reviews and approves the Charter;
- Ensures that those involved in the day-to-day management of the study are excluded from DSMB voting procedures;
- Discusses DSMB recommendations with RESPOND Study Management Team and other appropriate members of the project team via teleconference; and
- Takes and maintains minutes from closed sessions of DSMB teleconferences until study termination when the minutes are transferred to the custody of RESPOND Study Management Team.

**MEETINGS OF THE DSMB**

Organisational Meeting

The first meeting of the DSMB will be an organizational meeting. This meeting will formally establish the DSMB and begin to acquaint the DSMB members with the protocol or types of protocols that this DSMB will be charged with monitoring. It affords the DSMB an opportunity to recommend final revisions to the Charter and the communication plan between the DSMB, the RESPOND Study Management Team.

Scheduled Protocol and Data Review Meetings

Each protocol and data review meeting may consist of three sessions: Open Session, Closed Session, and Closed Executive Session.

Open Session

This will begin with an introductory session that includes introductions, roll call, assurance of a quorum, a reminder about the confidential nature of the proceedings and corresponding documentation, and a review of conflict of interest for all DSMB members.

Following the introductory session, the DSMB will move into the open session. Attendees will include the DSMB members, voting and *ex officio* members, the RESPOND Coordinating Principal Investigator and other study staff personnel, and appropriate RESPOND Trial Steering Committee members. This session may also be open to others as appropriate and upon invitation.

The open session will serve as a general study update. The RESPOND Coordinating Principal Investigator will be called upon to present study status and known relevant findings. Others with specific safety experience or concerns may also be called upon to present. The session will provide a forum for an exchange of information among the various groups involved in the conduct of the study. It will afford the DSMB members an opportunity to question the project team about the study and to seek additional information deemed relevant to the data review. Discussions may include progress of the study, including adverse events, disease status of participants, comparability of groups with respect to baseline factors, protocol compliance, site performance, quality control, and timeliness and completeness of follow-up. Only masked data will be reviewed and/or discussed during the open session.

Closed Session

Following the open session of the meeting, a closed session involving the DSMB members will be held to review unmasked and/or grouped safety data, discuss findings, and/or develop and vote on recommendations. During this session, any issues related to subject safety will be discussed. Requests by DSMB Members for the unmasking of data may be made at this time or prior to the meeting. An unmasked statistician should be available to provide guidance and answer questions.

Closed Executive Session

A brief teleconference may be held between the DSMB Chair and the specified RESPOND Study Management Team to discuss the recommendations of the DSMB.

Unscheduled Meetings/Reports

Unscheduled meetings can be requested by any party with the responsibility of overseeing the study.

**COMMUNICATION**

Reports to the DSMB

Associated SAEs and AEs will be provided to the DSMB yearly or as requested by the DSMB Chair. Study status reports will be provided to the DSMB at least two weeks prior to each scheduled meeting.

DSMB Minutes

The DSMB meetings may be recorded for the purpose of documenting meeting minutes. Once the Chair approves the minutes, the recordings will be destroyed.

The RESPOND Study Management Team will prepare the draft meeting minutes of the open session and forward to the DSMB Chair, RESPOND Study Management Team for review within one week following the DSMB meeting. Minutes of the open session will describe the proceedings. Draft minutes will be distributed to named attendees for review and comment.

Minutes of the closed session will describe the proceedings of the closed session. Minutes will be taken by the DSMB Chair. If unmasked information is reviewed during the closed session, minutes containing unmasked information will be marked as “Confidential” and distributed to the members of the DSMB only.

At the conclusion of the study, a complete set of the minutes of the closed sessions and the closed reports will be sent to RESPOND Study Management Team.

Recommendations

Following the closed session, a brief teleconference will be held between the DSMB Chair, RESPOND Study Management Team to discuss the recommendations of the DSMB.

The DSMB can recommend to the RESPOND Study Management Team that the current study continue without modification, continue with specified modifications, discontinue one or more arms of the study, or halt or modify the study until more information is available.

**BIBLIOGRAPHY**

Department of Health, Education, and Welfare, Office of the Secretary, Protection of Human Subjects. Belmont Report: Ethical Principles and Guidelines for the Protection of Human Subjects of Research, Report of the National Committee for the Protection of Human Subjects of Biomedical and Behavioral Research. DHEW Publication No. (OS) 78-0013 and No. (OS) 78-0014. 18 April 1979.

Ellenberg, Susan S., Fleming, Thomas R., DeMets, David L. Data Monitoring Committees in Clinical Trials, A Practical Perspective. John Wiley and Sons, LTD, West Sussex, England, 2002.

The Greenberg Report (1988). Organization, review, and administration of cooperative studies: a report from the heart special project committee to the National Advisory Heart Council. Controlled Clinical Trials 9 (2): 137-148.

US Food and Drug Administration (2006) Guidance for Clinical Trial Sponsors on the Establishment and Operation of Clinical Trial Data Monitoring Committees. Rockville, MD: FDA. <http://www.fda.gov/RegulatoryInformation/Guidances/ucm127069.htm>.

UNICEF/UNDP/World Bank/WHO (31 March 2004). Operational Guidelines for the Establishment and Functioning of Data and Safety Monitoring Boards. Geneva, Switzerland.

National Institute of Health (May 1, 2001). Policy and Guidelines for Data and Safety Monitoring.

DAMOCLES Study Group (2005). A proposed charter for clinical trial data monitoring committees; helping them do their job well. Lancet 365:711-22.

**S5. Description of data sources and data monitoring plan for the RESPOND trial**

**DATA SOURCES**

Two data sources are being used for data collection:

- Clinical record: Data are recorded from the patient hospital medical record including demographic variables, comorbid status, type of infection, severity at baseline, primary endpoints, secondary endpoints, pre-determined physiological, biochemical and microbiological variables of interest, safety, and long-term outcome measures. Administration of study drugs, duration, doses, amount of drug received, adverse events, protocol deviations and reason for protocol violations are also recorded; and
- Parent-completed follow-up questionnaires: Parent-reported screening questionnaires using validated tools collect child and parent outcomes. Questionnaires are completed at baseline and six months post-randomisation.

Data from both sources are collected into the purpose-built study database developed in Research Electronic Data Capture (REDCap), hosted by The University of Queensland (1, 2), by direct entry. The follow-up questionnaires for collecting long-term outcome measures are administered by a secure web link provided to parents in an email, with responses collected in the study database. In the case of parent comorbidity or circumstances limiting completion of the online screeners, questionnaires will be administered via telephone interview by the site Research Coordinator.

**DATA MONITORING PLAN**

Data monitoring is being performed throughout the trial, based on a data monitoring plan devised by the study team. The data monitoring plan utilises a risk-based approach which is in accordance with the ICH E6 (R2) Good Clinical Practice Guideline (3) and reflects current best practice for data monitoring practices in investigator-initiated trials. Briefly, the data monitoring plan includes the following components:

- Onsite and/or remote monitoring to conduct source data verification on 100% of data items relating to screening eligibility, randomisation, consent, study treatments, and those required for the calculation of the primary outcome and key secondary outcomes for every enrolled patient. Source data verification will also be performed on data items relating to the calculation of remaining secondary outcomes and key cohort descriptors on a random sample of 10% of enrolled patients from each site; and
- Centralised monitoring to evaluate recruitment statistics; evaluate completeness and rates of critical data within and across study sites; and review automated range and logic discrepancies from data quality rules in the REDCap study database.

The original study REDCap study database was enhanced to facilitate the activities outlined in the data monitoring plan. An independent monitor for each site will perform on-site monitoring, or remote monitoring utilising the institutional programme to share desktop computer screens or remote access to electronic medical records. Once data monitoring is finalised, the patient’s REDCap data entry record is locked in preparation for analysis. Site visits, independent audits, and regular monitoring of the blood sample storage will also be performed. At completion of the study, the monitor will ensure that there are plans for long-term storage of all relevant data and source documentation.

**REFERENCES**

1. Harris PA, Taylor R, Minor BL, Elliott V, Fernandez M, O’Neal L, McLeod L, Delacqua G, Delacqua F, Kirby J, Duda SN, Consortium, R.E. The REDCap consortium: building an international community of software platform partners. J Biomed Inform. 2019;95:103208.
2. Harris PA, Taylor R, Thielke R, Payne J, Gonzalez N, Conde JG. Research Electronic Data Capture (REDCap)–a metadata-driven methodology and workflow process for providing translational research informatics sup port. J Biomed Inform. 2009;42(2):377–81.
3. International Council for Harmonisation of Technical Requirements for Pharmaceuticals for Human Use. ICH Harmonised Guideline: Integrated Addendum to ICH E6 (R1): guideline for good clinical practice E6 (R2). 2016.

**S6. Listing of protocol deviations recorded for the RESPOND trial**

*Protocol deviations listed with a * contribute to exclusions applied to participants to determine the per-protocol cohort. ^#^ indicates protocol deviations will be reviewed on a case-by-case basis for applicability to per-protocol cohort selection.*

A consultant or investigator initiated withdrawal from the study prior to the finalisation of informed consent

Time taken to obtain informed consent exceeded 24 hours

Informed consent obtained but no study data collected

Informed consent obtained but patient was not ultimately randomised

Informed consent implemented / attempted but patient did not meet study specified inclusion / exclusion for enrolment in the study

*Patient randomised but there was no intention to treat

*Patient randomised but did not meet study specified inclusion / exclusion criteria for enrolment in the study

Patient randomised but informed consent was ultimately not obtained

*Patient randomised to an incorrect strata

*Patient randomised but did not receive / commence on any study treatment(s)

Patient randomised while study on hold

*First treatment received / commenced is not the same as the randomised allocation

*Study treatment not delivered according to protocol

*Patient randomised to "Vitamin C and Hydrocortisone" but did not receive Vitamin C

*Patient randomised to "Vitamin C and Hydrocortisone" but did not receive Hydrocortisone

*Patient randomised to "Hydrocortisone only" but received Vitamin C

*Patient randomised to "Hydrocortisone only" but did not receive Hydrocortisone

*Patient randomised to "Standard Care" but received Vitamin C

Protocol not followed for blood sampling

Baseline survey not provided by research coordinator

6-month follow-up not attempted by research coordinator

*Trial drugs continued >24 hours post ceased inotropes

*Clinical decision to cease trial drugs < 3 days while still being treated for septic shock

*^#^Protocol not followed

*^#^Other

**Figure S1.** Proposed participant flow diagram including long-term outcomes


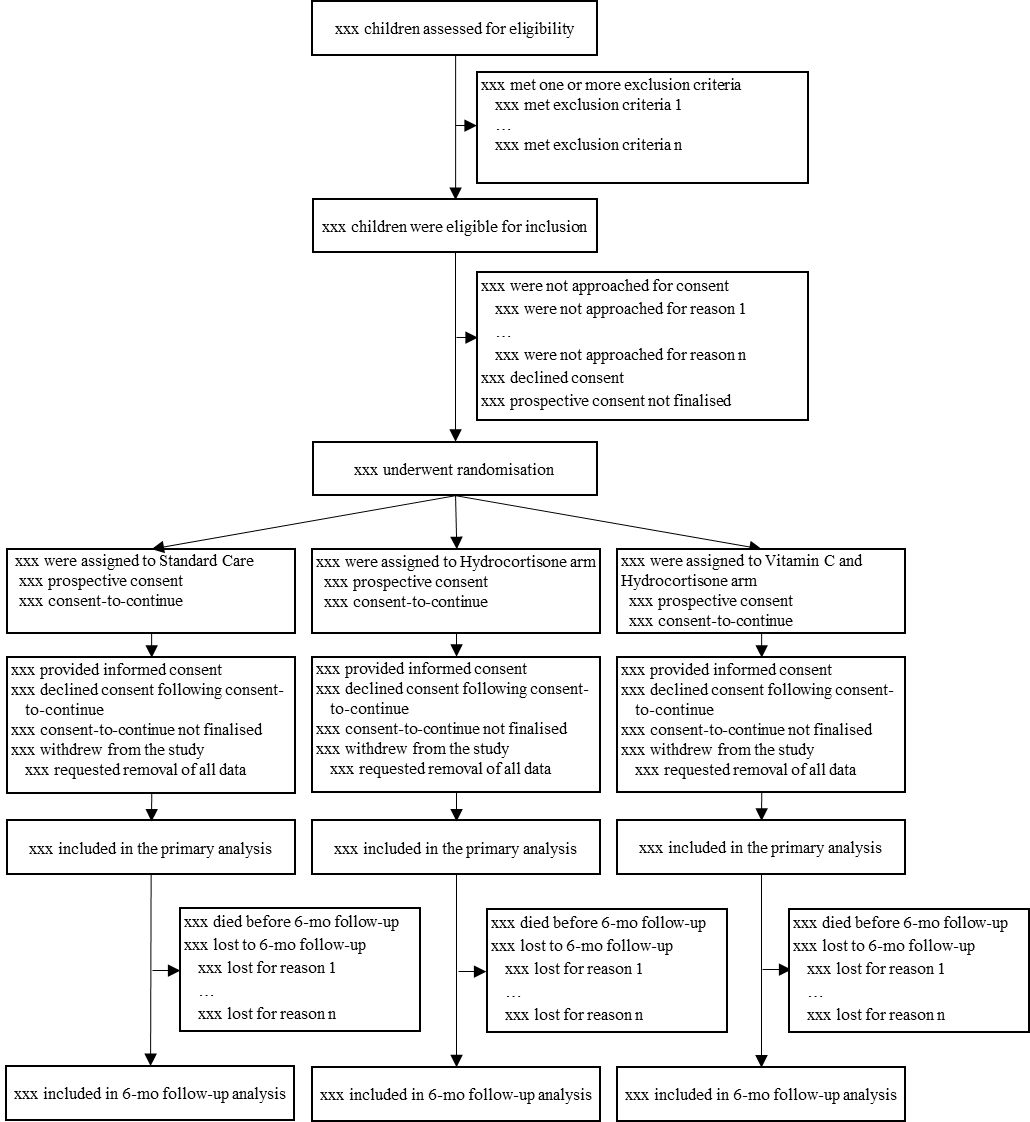


**Table S1. Definition of primary and secondary outcome measures**

| **Outcome** | **Definition** |
| --- | --- |
| *Short-term outcomes* | |
| Time alive and free of inotropes/vasopressors *(primary outcome)* | Defined as the duration in days alive and free of inotropes/vasopressors (dopamine, dobutamine, adrenaline, noradrenaline, milrinone, or vasopressin) in the first 7 days post randomisation; patients dying within 7 days of randomisation will be assigned a value of zero. The following continuous infusions will be considered as inotropes/vasopressors: adrenaline, noradrenaline, dopamine, dobutamine, vasopressin, milrinone. |
| Alive and free of multi-organ dysfunction at 72 hours post-randomisation | Composite binary outcome measure of multi-organ dysfunction and survival status at 72 hours post-randomisation. Multi-organ dysfunction is defined as a pSOFA* (pediatric Sequential Organ Failure Assessment) organ-specific subscore increase in at least 2 organs from baseline within 72 hours of randomisation. Patients discharged from PICU prior to 72 hours post-randomisation will be considered to have no organ dysfunction at the 72 hour timepoint, presuming the patient is alive. Denominator is number of enrolled patients. |
| PICU-free survival | Duration of days alive and free from PICU admission, censored at 28 days post-randomisation. Patients dying within 28 days of randomisation will be censored as zero days to correct for the competing effect of mortality on PICU length of stay. Length of PICU stay for patients re-admitted to PICU within the same hospital admission and within 28 days of randomisation will be added to the index length of stay. |
| Survival free of organ support | Duration of days alive and free from invasive ventilation, inotropes/vasopressors, extracorporeal membrane oxygenation (ECMO), and renal replacement therapy, censored at 28 days post-randomisation. Patients dying within 28 days of randomisation will be recorded as zero days. |
| Survival free of cardiovascular support | Duration of days alive and free from inotropes/vasopressors and ECMO, censored at 28 days post-randomisation. Patients dying within 28 days of randomisation will be recorded as zero days. |
| Survival free of ventilation | Duration of days alive and free from invasive ventilation, censored at 28 days from randomisation. Patients dying within 28 days of randomisation will be recorded as zero days. |
| Day 28 mortality | Death occurring within 28 days of randomisation. Denominator is number of enrolled patients. |
| PICU length of stay | Measured as days from randomisation to PICU discharge, censored at 28 days post-randomisation. Deaths occurring within 28 days post-randomisation will be censored at time of death. Length of PICU stay for patients re-admitted to PICU within the same hospital admission and within 28 days of randomisation will be added to the index length of stay. |
| Hospital length of stay | Measured as days from randomisation to hospital discharge, censored at 28 days post-randomisation. Only the index hospital admission will be included (i.e. if there are subsequent hospital admissions within 28 days post-randomisation, the length of stay of such admissions will not contribute to the outcome). Deaths occurring within 28 days post-randomisation will be censored at time of death. |
| Hyperglycaemia within 7 days of randomisation | Treated with intravenous insulin for high blood glucose or higher than usual requirement of insulin dose (if a known diabetic) within 7 days of randomisation. |
| Hypoglycaemia within 7 days post-randomisation | Blood glucose level <2.6 mmol/L within 7 days post-randomisation. |
| Hypernatraemia within 7 days post-randomisation | Serum sodium >155 mmol/L within 7 days post-randomisation |
| Hospital-acquired microbiologically confirmed infection within 28 days post-randomisation | Hospital-acquired microbiologically confirmed infection within 28 days post-randomisation. |
| Oxalate nephropathy within 28 days post-randomisation | As reported by treating medical team within 28 days post-randomisation |
| Haemolysis within 7 days post-randomisation | As reported by the treating medical team within 7 days post-randomisation, and if not explained by other factors such as ECMO. |
| Worsening of liver function within 7 days post-randomisation | >2x increase in Alanine Transferase (ALT) from baseline to day 7 post-randomisation and/or an increase in total bilirubin of more than 68 micromol/L from baseline to day 7 post-randomisation. |
| Costs | Total direct healthcare costs for the index hospital admission, including PICU and ward bed-days, organ-support therapies, investigations, and medication use. Costs will be derived using standardised unit cost estimates for Australian sites. |
| *Long-term outcomes* | |
| Health-related quality of life | Utility weight at 6 months derived from the Pediatric Quality of Life Inventory (PedsQL) scores using a validated mapping algorithm. |
| Quality-adjusted life years (QALYs) | Patient-level QALYs calculated from survival and health state utilities between randomisation and 6 months post-randomisation. |
| Total healthcare costs to 6 months | Sum of index hospitalisation costs and post discharge healthcare utilisation collected at 6-month follow-up. |
| Functional status 6 months post-randomisation | 1. Change in Functional Status Score (FSS) from baseline to 6 months post-randomisation. Participants without a recorded FSS at baseline but who have a score at 6 months will not be included in the calculation of the outcome measure, but will have their 6 month FSS reported descriptively. 2. Modified Paediatric Overall Performance Category^ (combined Paediatric Overall Performance Category and Modified Glasgow Outcome Score) at 6 months post-randomisation, with categories grouped as good/normal/functionally normal, mild disability, moderate/severe disability, coma/vegetative state, brain death. |
| Neurodevelopmental vulnerability at 6-months post-randomisation | A binary indicator of impairment if, in any of the following assessments relevant for age of the child, impairment is indicated (identified as developmental assessment >1 SD below the normative mean, or exceeding specified impairment cutoffs, as per assessment guidance):   - Pediatric Quality of Life Inventory (PedsQL) Total Score; - Ages and Stages Questionnaire, 3rd Edition (ASQ-3) Domain Scores OR Strengths and Difficulties Questionnaire (SDQ) Total Difficulties Score; - Pediatric Emotional Distress Scale (PEDS) Total Score OR Children’s Revised Impact of Events Scale (CRIES-13) Total Score; - Behavior Rating Inventory for Executive Function (BRIEF-2) Global Executive Composite Score OT Behavior Rating Inventory for Executive Function for Pre-schoolers (BRIEF-P) Global Executive Composite Score.   Assessments are completed in the order presented in the preceding sentence, aligned with order of importance. The primary analysis will use only complete assessment data (i.e. completion of all age relevant assessments). A sensitivity analysis will include patients with at least PedsQL completed, assuming that if a participant does not have complete data for any individual assessment, there will be no vulnerability assumed in that domain. Individual components of this outcome will also be presented; this is particularly relevant for PedsQL which was the only tool completed by participants recruited from Brazilian sites. |

**Matics TJ, Sanchez-Pinto LN. Adaptation and validation of a pediatric sequential organ failure assessment score and evaluation of the sepsis-3 definitions in critically ill children. JAMA Pediatrics. 2017 Oct 1;171(10):e172352)*.

^ *Schlapbach LJ, Horton SB, Long DA, Beca J, Erickson S, Festa M, d’Udekem Y, Alphonso N, Winlaw D, Johnson K, Delzoppo C. Study protocol: NITric oxide during cardiopulmonary bypass to improve Recovery in Infants with Congenital heart defects (NITRIC trial): a randomised controlled trial. BMJ open. 2019 Aug 1;9(8):e026664; Gibbons KS, Schlapbach LJ, Horton SB, Long DA, Beca J, Erickson S, Festa M, d’Udekem Y, Alphonso N, Winlaw D, Johnson K. Statistical analysis plan for the NITric oxide during cardiopulmonary bypass to improve Recovery in Infants with Congenital heart defects (NITRIC) trial. Critical Care and Resuscitation. 2021 Mar 1;23(1):47-58.*
